# Therapeutic Potential of Adipose Stem Cell-Derived Conditioned Medium on Scar Contraction Model

**DOI:** 10.3390/biomedicines10102388

**Published:** 2022-09-24

**Authors:** Yukiko Imai, Nobuhito Mori, Yuma Nihashi, Yutaro Kumagai, Yoichiro Shibuya, Junya Oshima, Masahiro Sasaki, Kaoru Sasaki, Yukiko Aihara, Mitsuru Sekido, Yasuyuki S. Kida

**Affiliations:** 1 Cellular and Molecular Biotechnology Research Institute, National Institute of Advanced Industrial Science and Technology (AIST), Tsukuba 305-8565, Ibaraki, Japan; 2 Department of Plastic and Reconstructive surgery, Faculty of Medicine, University of Tsukuba, Tsukuba 305-8575, Ibaraki, Japan; 3 School of Integrative and Global Majors, University of Tsukuba, Tsukuba 305-8575, Ibaraki, Japan

**Keywords:** scar contracture, pathological scar, scar contraction model, fibroblast, adipose mesenchymal stem cell

## Abstract

Scars are composed of stiff collagen fibers, which contract strongly owing to the action of myofibroblasts. To explore the substances that modulate scar contracture, the fibroblast-populated collagen lattice (FPCL) model has been used. However, the molecular signature of the patient-derived FPCL model has not been verified. Here, we examined whether the patient-derived keloid FPCL model reflects scar contraction, analyzing detailed gene expression changes using comprehensive RNA sequencing and histological morphology, and revealed that these models are consistent with the changes during human scar contracture. Moreover, we examined whether conditioned media derived from adipose stem cells (ASC-CM) suppress the scar contracture of the collagen disc. Detailed time-series measurements of changes in disc area showed that the addition of ASC-CM significantly inhibited the shrinkage of collagen discs. In addition, a deep sequencing data analysis revealed that ASC-CM suppressed inflammation-related gene expression in the early phase of contraction; in the later phase, this suppression was gradually replaced by extracellular matrix (ECM)-related gene expression. These lines of data suggested the effectiveness of ASC-CM in suppressing scar contractures. Therefore, the molecular analysis of the ASC-CM actions found in this study will contribute to solving medical problems regarding pathological scarring in wound prognosis.

## 1. Introduction

A scar is a tissue composed of stiff collagen fibers, and it becomes the final form of wound healing in cases where intact tissue is deeply injured. After skin injury, the wound contracts strongly, thereby reducing the wound area. This contraction is caused by the action of myofibroblasts derived from skin fibroblasts and the extracellular matrix regulated by fibroblasts [1,2]. Excessive contraction often causes skin tightness called scar contracture [3]. Indeed, the stronger the contractile force, the more stress applied to the wound, which can lead to pathological scarring. Typically, fibroblasts that are induced at the injury site undergo apoptosis after wound healing [4]; however, their continued abnormal proliferation, together with abnormal collagen production, leads to the formation of pathological scars, such as keloids and hypertrophic scars, which can cause functional and esthetic issues [5]. More specifically, keloids are caused by chronic inflammation in the reticular dermis, which is triggered by physical stress at the wound site, resulting in the sustained proliferation and concurrent reduction in the apoptotic function of fibroblasts. Moreover, keloids and hypertrophic scars are histologically characterized by the formation of thick collagen fiber bundles [6]. Hence, the myofibroblast marker alpha-smooth muscle actin (α-SMA, *ACTA2*), as well as collagen 1 and collagen III, and the inflammation-related cytokines interleukin (IL)-6 and transforming growth factor-beta (TGF-β) are highly expressed in keloids and hypertrophic scars [7,8,9]. The fibroblast-populated collagen lattice (FPCL) is a classical wound repair model developed by Bell et al. in 1979. By embedding fibroblasts into a collagen gel, this experimental model causes the gel to take the form of a disc. Subsequently, the collagen discs significantly decrease in diameter over time and become dense and constricted [10]. This phenomenon is considered a simple model of the in vitro contraction of connective tissues that occurs during wound healing to quantify the contractility of fibroblasts [11,12,13,14]. While this simple in vitro model may be useful in exploratory studies to identify substances and molecules that suppress scar contracture, detailed histological and gene expression analyses are lacking.

Surgical removal is the mainstay of treatment for pathological scars. However, due to the invasive nature of surgery and the associated risk of recurrence, it is not an acceptable treatment for all patients. Therefore, non-surgical methods for treating pathological scars have recently been explored [15]. In this context, research targeting fibroblasts in scar tissue has been actively conducted, targeting their ability to synthesize extracellular matrix and suppress fibroblast proliferation [16,17]. One of these studies recently focused on using mesenchymal stem cells (MSCs) as a material for scar therapy [18]. Adipose-derived stem cells (ASCs) are a type of MSC abundant in adipose tissue. They have been attracting attention as a source of MSCs because of their advantage of being easy to collect and having characteristics comparable to those of MSCs [19].

This study analyzed the global trends of gene expression during wound contraction using a FPCL model and examined whether the patient-derived FPCL model could be used as a human scar contraction model. Moreover, the suppressive effects of supernatants derived from ASCs on scar contracture were examined using this verified model, and their efficacy on scar formation was analyzed. The biological analysis of the efficacy of ASC supernatant in this study is expected to contribute to developing technology for regenerative medicine and solving the functional and aesthetic problems associated with pathological scars in wound prognosis.

## 2. Materials and Methods

### 2.1. Fibroblasts Isolation and Culture

Fibroblasts were donated by patients who were clinically diagnosed with keloids or hypertrophic scars and were to undergo surgical resection. This study was approved by the Ethics Committee of the University of Tsukuba Hospital. Approval was obtained from the Tsukuba Clinical Research and Development Organization (T-CReDO; protocol number: H30-186). Written informed consent was obtained from all patients to provide keloid specimens for research purposes.

Keloid fibroblasts (KFs) were established from surgical specimens using the explant method, as described previously [20]. Briefly, fresh keloids obtained during surgical excision were washed thrice in phosphate-buffered saline (PBS; FUJIFILM Wako Pure Chemical Corporation, Osaka, Japan) containing 1% penicillin-streptomycin solution (FUJIFILM Wako Pure Chemical Corporation Osaka, Japan). The specimens were then dissected free of fat and epidermis, minced into 2-mm pieces, and attached to a 60-mm cell culture dish that was specially treated for cell culture (CellBIND; Corning, NY, USA). Cells were grown in Dulbecco’s modified Eagle’s medium (DMEM; FUJIFILM Wako Pure Chemical Co., Richmond, VA, USA) supplemented with 10% fetal bovine serum (FBS; Thermo Fisher Scientific Inc., Waltham, MA, USA) and 1% penicillin-streptomycin solution for the primary culture. The fibroblasts were retrieved after 21–24 days of incubation at 37 °C and 5% CO_2_, and cell passages were performed. The fibroblasts passaged 2–4 times were used in the present study.

### 2.2. Fibroblast Populated Collagen Lattice (FPCL) Contraction Assay

**Figure 1:**
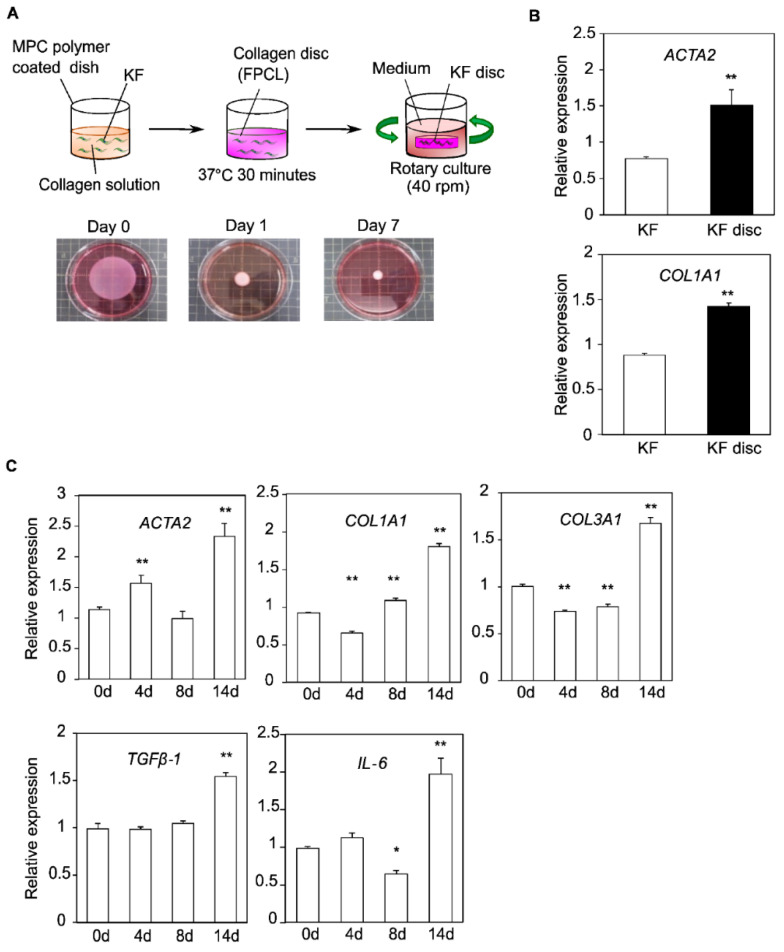
Construction of FPCL contraction model. (**A**) (Above) Schematic representation of establishing FPCL contraction model. (Below) Gel contraction is presented by images of KF discs. (**B**) qPCR analysis of *ACTA2* and *COL1A1* in monolayer cultured KF and KF disc. Results are presented as the mean ± standard deviation of three biological replicates. ** *p* < 0.01, unpaired Student’s *t*-test. (**C**) Changes over time in gene expression of scar markers in KF discs. Results are presented as the mean ± standard deviation of three biological replicates. * *p* < 0.05, ** *p* < 0.01 compared with day 0 gene expression, one-way analysis of variance followed by Tukey’s multiple method. Abbreviations: FPCL, fibroblast-populated collagen lattice; KF, keloid fibroblasts; qPCR, quantitative real-time polymerase chain reaction; MPC, 2-methacryloyloxyethyl phosphorylcholine.

The fibroblast-populated collagen lattice (FPCL) was prepared as described previously (Figure 1A) [21]. Briefly, keloid fibroblasts were dissociated from culture dishes and suspended in neutralized type I collagen solution (IAC-30, Koken Co., Tokyo, Japan) at a density of 1 × 105 cells/mL. The collagen solution was poured into the 24-well tissue culture plate (Corning, NY, USA) coated with 2-methacryloyloxyethyl phosphorylcholine polymer (LIPIDURE-CM5206, NOF Co., Tokyo, Japan) at a volume of 0.8 mL/well. After 30 min of incubation at 37 °C for gelation, the gels were released from their wells using a vortex mixer (GENIE 2; Scientific Industries Inc., Bohemia, NY, USA) and 1 mL of medium was poured into each well. The gel contraction was calculated as the percentage of the lattice area to the initial gel area using imaging software, ImageJ (1.53c, Java 1.8.0_172, U. S. National Institutes of Health, Bethesda, MD, USA) [22].

### 2.3. The Preparation of Adipose Derived Mesenchymal Stem Cell-Conditioned Medium (ASC-CM)

The human immortalized ASC cell line ASC52telo (SCRC-4000; ATCC, Manassas, VA, USA) was used for ASC-CM extraction. SCRC-4000 cells were cultured in DMEM supplemented with 10% FBS and 1% penicillin-streptomycin until they reached confluence. The waste culture medium was removed, and the cells were washed twice in PBS. Subsequently, fresh serum-free DMEM was added to the cells. The cells were then incubated at 37 °C in a humidified atmosphere containing 5% CO_2_ for 48 h. After 48 h of incubation, the medium was collected and ultra-filtrated with a 0.45 µm filter. The supernatant obtained after filtering the cell components was used as the ASC-CM without further dilution. FBS was then added to achieve a concentration of 10% when used for experiments.

### 2.4. Quantitative Real-Time Polymerase Chain Reaction (qPCR)

Total ribonucleic acid (RNA) was isolated from the cells using NucleoSpin RNA (Macherey Nagel GmbH & Co., KG, Duren, Germany) and from the collagen discs using ISOGEN reagent (Nippon Gene; Tokyo, Japan), according to the manufacturer’s instructions. Subsequently, 100 ng of total RNA was reverse-transcribed into complementary deoxyribonucleic acid (cDNA) using ReverTra Ace reverse-transcription reagents (TOYOBO, Osaka, Japan), according to the manufacturer’s instructions. A qPCR was performed using the LightCycler 96 System (Roche, Basel, Switzerland) with the THUNDERBIRD SYBR qPCR Mix (TOYOBO, Osaka, Japan). The qPCR data were analyzed using the delta-delta cycle threshold method with U36b4 as a housekeeping gene and are shown as the mean ± standard deviation (SD) of triplicate measurements.

The primer sequences used are as follows: *ACTA2*, (forward) 5′-GAGGCACCCCTGAACCCCAA-3′, (reverse) 5′-ATCTCCAGAGTCCAGCACGA-3′; COL1A1, (forward) 5′-TGACCTCAAGATGTGCCACT-3′, (reverse) 5′-ACCAGACATGCCTCTTGTCC-3′; COL3A1, (forward) 5′-GCTGGCATCAAAGGACATCG-3′ (reverse) 5′-TGTTACCTCGAGGCCCTGGT-3′; *TGFβ-1*, (forward) 5′-GCAGCACGTGGAGCTGTA-3′, (reverse) 5′-CAGCCGGTTGCTGAGGTA-3′; *IL-6*, (forward) 5′-GGTACATCCTCGACGGCATCT-3′, (reverse) 5′-GTGCCTCTTTGCTGCTTTCAC-3′; *U36b4*, (forward) 5′-AGATGCAGCAGATCCGCA-3′ (reverse) 5′-GTTCTTGCCCATCAGCACC-3′.

### 2.5. Gene Expression Analysis (RNA-Sequencing)

RNA-seq libraries were prepared using TruSeq Stranded messenger ribonucleic acid (mRNA) (Illumina, San Diego, CA, USA), and sequencing was performed using a NovaSeq 6000 (Illumina). The acquired data from each time point for each condition were mapped and quantified using STAR (2.7.1a, ColdSpring Harbor Laboratory, Cold Spring Harbor, NY, USA) [23] and RSEM (1.3.1, Universityof Wisconsin-Madison, Madison, WI, USA) [24] with hg38 as the reference genome and Ensemble GRCh38 as the gene annotation. Differentially expressed genes were analyzed using edgeR (3.24.3, Walter and Eliza HallInstitute of Medical Research, Victoria, Australia) [25,26] in R (3.5.1, R Foundation for StatisticalComputing, Vienna, Austria) [27]. A Gene Ontology (GO) enrichment analysis was performed using the Database for Annotation, Visualization, and Integrated Discovery (DAVID) [28,29] through RDAVID WebService (1.20.0, Catholic University of C´ordoba, C´ordoba, Argentina.) [30] and visualized using clusterProfiler (3.10.1, Southern Medical University, Guangzhou, China) [31]. A Gene Set Enrichment Analysis (GSEA) was performed using the GSEA software (GSEA software 4.2.3, Broad Institute, Cambridge, MA, USA) available on the GSEA-Broad Institute website [32,33]. A GO gene set library (c5.go.bp.v7.5.1.symbols.gmt, Frederick National Laboratory for Cancer Research Frederick, MD, USA) was used to analyze the expression data. Gene sets with a false discovery rate (FDR) of < 0.05 and a nominal *p* value of < 0.05 were considered significant.

### 2.6. Histological Analysis

All staining procedures were performed on 4 µm-thick sections. Hematoxylin and eosin (HE) staining and Masson’s trichrome (MT) staining were performed according to standard protocols. The amount of collagen was measured by performing a threshold process in ImageJ (1.53c, Java 1.8.0_172, U. S. National Institutes of Health, Bethesda, MD, USA) [22] and calculating its intensity.

### 2.7. Statistical Analysis

Data are represented as mean ± standard deviation (SD). The Student’s *t*-test was used to determine the significance of the differences between the two groups. A one-way analysis of variance (ANOVA) followed by Tukey’s honestly significant difference test was used to test three or more independent experimental samples. All statistical analyses were performed using EZR (Saitama Medical Center, Jichi Medical University, Saitama, Japan), which is a graphical user interface for R (The R Foundation for Statistical Computing, Vienna, Austria) [34]. Statistical significance was set at *p* < 0.05. Error bars indicate SD.

## 3. Results

### 3.1. Construction of the FPCL Model

KFs were cultured in a monolayer from scar tissue collected from patients, and these cultured cells were embedded in collagen gel and cured to form collagen discs (KF discs). Later, the collagen discs were shrunk by rotary culture and cultured for seven days (Figure 1A). The expressions of *ACTA2* and *COL1A1*, known to be upregulated in scar tissue, were compared using a qPCR to check the maturation of the KF-derived FPCL model. The results showed that the expression of both *ACTA2* and *COL1A1* was significantly upregulated in KF discs compared to that in monolayer KF (Figure 1B).

Next, we examined changes in gene expression during the production of collagen discs. Changes in *ACTA2*, *COL1A1*, *COL3A1*, *TGFβ-1*, and *IL-6* were evaluated by a qPCR on days 0, 4, 8, and 14. The results show that the expression of these genes increased after 14 days of culture. Notably, the expression of *ACTA2* increased rapidly in the early stage of culture and reached its highest level on day 14 (Figure 1C).

### 3.2. Comprehensive Gene Expression Analysis in the Collagen Discs

**Figure 2:**
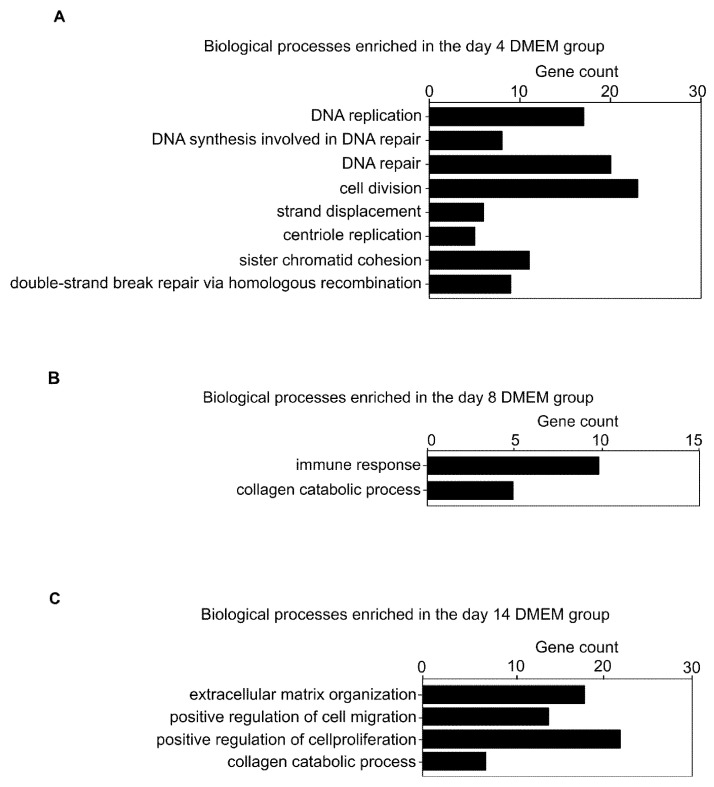
Global gene expression patterns during the production of the FPCL model. (**A**) GO terms on day 4. (**B**) GO terms on day 8. (**C**) GO terms on day 14. Abbreviations: FPCL, fibroblast-populated collagen lattice; GO, Gene Ontology; DNA, deoxyribonucleic acid; DMEM, Dulbecco’s modified Eagle’s medium; ECM, extracellular matrix.

The top 500 genes in the z-score of expression level for each day were extracted and subjected to a GO analysis to examine the gene expression changes during collagen disc formation. The GO terms are shown in Figure 2. On day 4, GO terms such as “DNA replication”, “DNA synthesis involved in DNA repair”, “DNA repair”, “cell division”, “strand displacement”, “centriole replication”, “sister chromatid cohesion”, and “double-strand break repair through homologous recombination” were prominent (Figure 2A, Table S1). On day 8, we found GO terms related to immunity and inflammation, such as “immune response” and “collagen catabolic process” (Figure 2B, Table S2). On day 14, we found ECM-related GO terms, such as “extracellular matrix organization” and “collagen catabolic process,” and GO terms related to cell migration and proliferation, such as “positive regulation of cell migration” and “positive regulation of cell proliferation” (Figure 2C, Table S3). Thus, gene expression changes in collagen discs are similar to those occurring in scar tissues in vivo [35].

### 3.3. The Similarity between Collagen Discs and Patient-Derived Scar Tissue in Histology and Gene Expression

**Figure 3:**
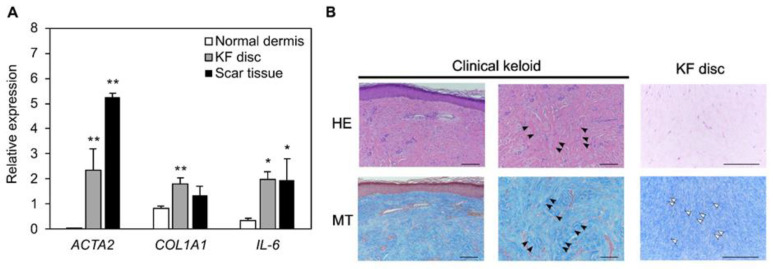
Comparison between collagen discs and scars in histology and gene expression. (**A**) qPCR analysis of scar markers in normal tissue, KF discs and scar tissue from patients. Results are presented as the mean ± standard deviation of three biological replicates. * *p* < 0.05, ** *p* < 0.01, compared with normal dermis gene expression, one way analysis of variance followed by Tukey’s multiple method. (**B**) Representative images of hematoxylin and eosin-stained and Masson’s Trichrome-stained clinical keloid tissue and KF disc sections are shown. Black arrows indicate the thick collagen fiber bundles in the deep dermis of keloid. White arrows indicate partial bundles of collagen discs. All bars indicate 200 μm. Abbreviations: qPCR, quantitative real-time polymerase chain reaction; KF, keloid fibroblasts.

A comprehensive gene expression analysis suggested that the induced global responses in the collagen disc were similar to those in human scar tissue. Keloid scar tissue was isolated from patients, and gene expression and histological similarities were examined to determine the similarities between the collagen discs and the human scar tissue. Additionally, the expression levels of *ACTA2* and *IL-6* were significantly upregulated by more than two-fold in scar tissues, compared with those in normal dermal tissues, indicating scar tissue-specific changes (Figure 3A).

Next, the histology between clinical keloids and collagen discs after shrinkage was compared using HE and MT staining, representing the collagen fibers (Figure 3B). In the human sample, scar tissue was characterized by the presence of thick collagen fiber bundles in the deep dermis (Figure 3B, black arrows). In contrast, the MT staining of collagen discs did not show large bundles of collagen fibrils compared to keloid tissue but showed partial bundles (Figure 3B, white arrows). Hence, collagen aggregation was also considered to be present in the FPCL model.

### 3.4. Suppression of the Collagen Disc Contraction by the ASC-CM

**Figure 4:**
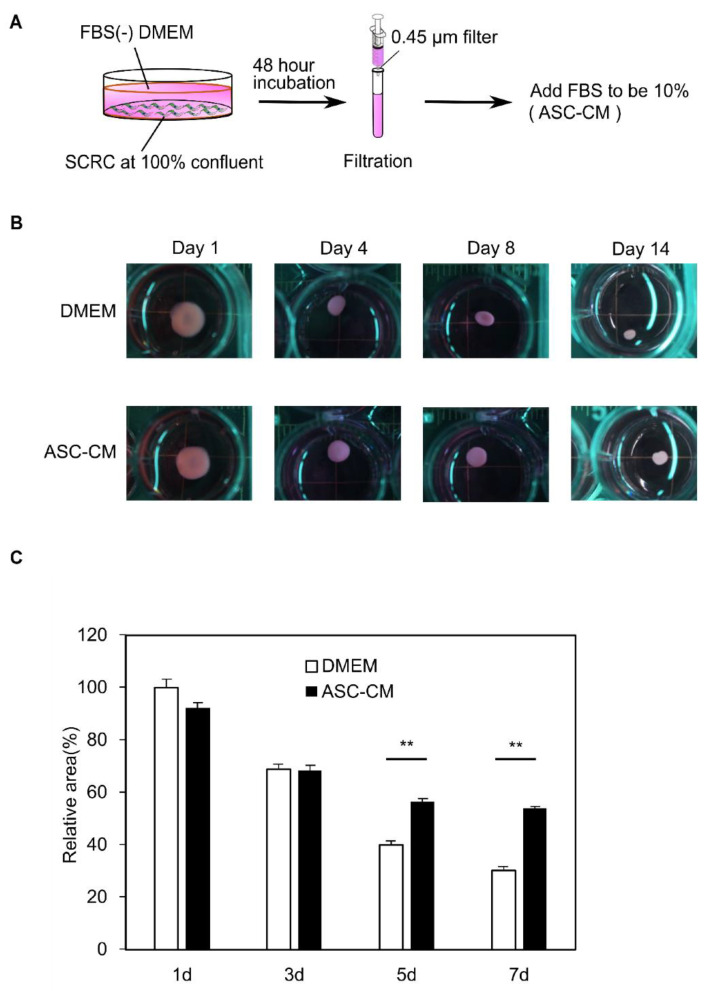
Suppression of shrinkage of KF disc by ASC-CM. (**A**) Schematic representation of ASC-CM preparation. (**B**) Images showed the difference in shrinkage of KF disc treated with DMEM or ASC-CM. (**C**) The relative contraction area was calculated as a percentage of the day 1 DMEM group. Results are presented as the mean ± standard deviation of six biological replicates. ** *p* < 0.01, unpaired Student’s *t*-test. Abbreviations: ASC-CM, conditioned media derived from adipose stem cells; KF, keloid fibroblasts; DMEM, Dulbecco’s modified Eagle’s medium; FBS, fetal bovine serum.

Next, we investigated whether ASC-CM affects the contraction of collagen discs. The preparation method for ASC-CM is shown in Figure 4A. The ASCs supernatant was collected using ultrafiltration and prepared as conditioned media with 10% FBS. To examine the effect of ASC-CM, the areas of KF collagen discs cultured in DMEM supplemented with 10% FBS were compared with those cultured in ASC-CM on days 1, 3, 5, and 7 (Figure 4B,C). Detailed time-series measurements of the change in disc area showed that collagen discs cultured in DMEM shrunk by 30% relative to day 1 after 7 days. In contrast, those cultured in ASC-CM shrunk by 58%, indicating that shrinkage was greatly mitigated by treatment with ASC-CM.

### 3.5. Changes in Histology and Gene Expression by the ASC-CM

**Figure 5:**
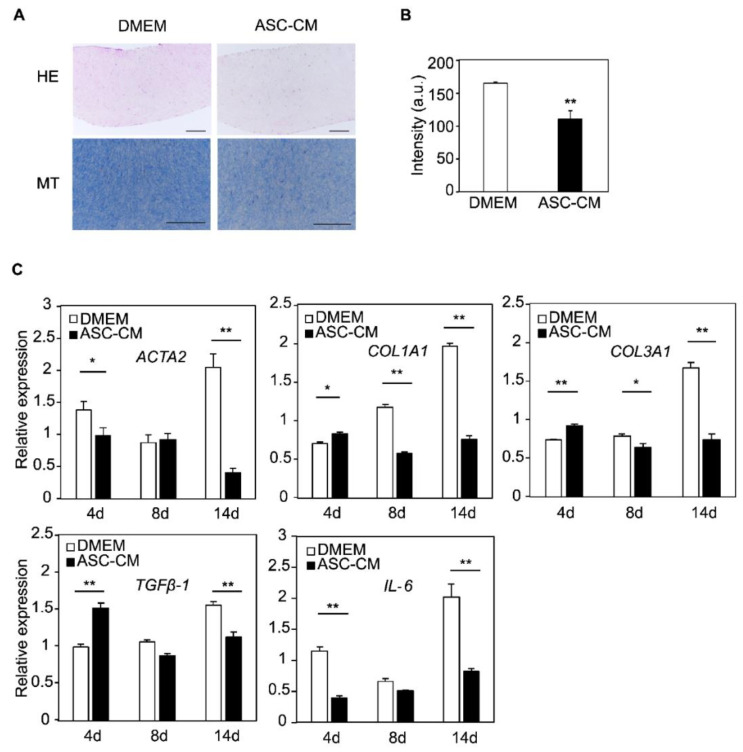
ASC-CM suppressed collagen aggregation and gene expressional changes in KF discs. (**A**) Representative images of hematoxylin and eosin-stained and Masson’s Trichrome-stained KF discs sections are shown. All bars indicate 200 μm. (**B**) Staining density of collagen fibers in KF disks. Results are presented as the mean ± standard deviation of three biological replicates. ** *p* < 0.01, unpaired Student’s *t*-test. (**C**) Gene expression analysis of scar markers in KF discs treated with DMEM or ASC-CM. Results are shown as relative gene expression on day 0. Results are presented as the mean ± standard deviation of three biological replicates. * *p* < 0.05, ** *p* < 0.01, unpaired Student’s *t*-test. Abbreviations: ASC-CM, conditioned media derived from adipose stem cells; KF, keloid fibroblasts; DMEM, Dulbecco’s modified Eagle’s medium; HE, hematoxylin and eosin; MT, Masson’s Trichrome.

We focused on the histological changes that occurred in ASC-CM-supplemented KF discs. The number of nuclei in the tissue sections of the KF disc was counted to examine the efficacy of cell proliferation during disc formation. The number of nuclei in the tissue section of the KF disc was 212 in DMEM and 120 in ASC-CM, indicating a decrease in the number of cells (Figure 5A). Next, collagen fibers were stained with Masson’s trichrome to examine the stromal mass. The results showed that the staining density of the collagen fibers in the ASC-CM cultures was reduced to 68% compared with that in the DMEM cultures (Figure 5B).

Changes in gene expression were also compared between the samples. As mentioned earlier, *ACTA2* was upregulated in the early stage of culture in the FPCL model; however, ASC-CM suppressed this rapid increase in *ACTA2* expression on day 4 and was pronounced on day 14. The expression of *COL1A1*, *COL3A1*, and *TGFβ-1* was also suppressed in the late stage of culture and was pronounced on day 14. In addition, *IL-6* was strongly suppressed during the early stages of culture. These gene expression trends suggest that ASC-CM suppressed the formation of collagen discs (Figure 5C).

### 3.6. Alteration of Global Trend of Gene Expression by the Addition of ASC-CM

We confirmed that ASC-CM regulates collagen disc contraction and biosynthesis by altering the expression of typical genes. A comprehensive mRNA expression analysis was performed to further analyze the global gene expression in detail. The transcriptome profiles of the DMEM- and ASC-CM-treated samples were compared each day.

**Figure 6:**
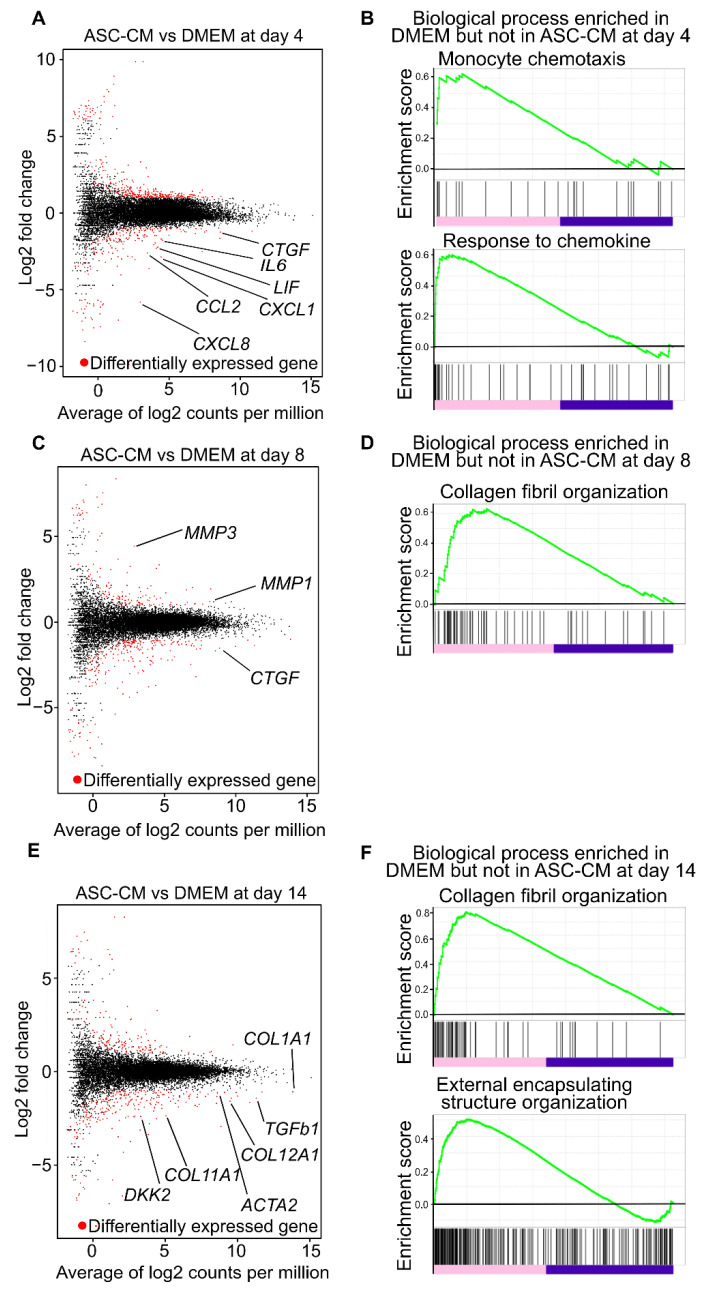
Global gene expression pattern in ASC-CM treatment. (**A**) MA plot, a scatter plot of log2 fold-change versus the average of log2 counts per million, showing differentially expressed genes as red points (adjusted *p* value < 0.05 and log2 [fold change] > 1) in ASC-CM treated KF discs compared to DMEM treated KF discs on day 4. (**B**) GSEA of DMEM treated KF discs and ASC-CM treated KF discs on day 4. Enrichment plots of expression signatures of “monocyte chemotaxis” and “response to chemokine”. (**C**) MA plot, a scatter plot of log2 fold-change versus the average of log2 counts per million, showing differentially expressed genes as red points (adjusted *p* value < 0.05 and log2 [fold change] > 1) in ASC-CM treated KF discs compared to DMEM treated KF discs on day 8. (**D**) GSEA of DMEM treated KF discs and ASC-CM treated KF discs on day 8. Enrichment plots of expression signatures of “collagen fibril organization”. (**E**) MA plot, a scatter plot of log2 fold-change versus the average of log2 counts per million, showing differentially expressed genes as red points (adjusted *p* value < 0.05 and log2 [fold change] > 1) in ASC-CM treated KF discs compared to DMEM treated KF discs on day 14. (**F**) GSEA of DMEM treated KF discs and ASC-CM treated KF discs on day 14. Enrichment plots of expression signatures of “collagen fibril organization” and “external encapsulating structure organization”. Abbreviations: ASC-CM, conditioned media derived from adipose stem cells; DMEM, Dulbecco’s modified Eagle’s medium; KF, keloid fibroblasts; GO, Gene Ontology; ECM, extracellular matrix; GSEA, Gene Set Enrichment Analysis.

On day 4, 1490 transcripts were differentially expressed, of which 868 were upregulated while 622 were downregulated in the ASC-CM-treated samples. Among those downregulated, *CXCL1*, *CCL2*, *CXCL8*, *CTGF*, *LIF*, and *IL-6* are known to be involved in fibrosis or cancer stroma [7,36,37,38,39] (Figure 6A). A GSEA was performed on all genes to identify pathways enriched in ranked gene lists. The results demonstrated that “monocyte chemotaxis” and “response to chemokine” were enriched in DMEM-treated samples but not in ASC-CM-treated samples (Figure 6B). In addition, the GO analysis of the top 10 lower *p*-values of the genes downregulated in the ASC-CM-treated samples showed inflammation-related terms, such as “immune response”, “chemokine-mediated signaling pathway”, “cellular response to tumor necrosis factor”, “inflammatory response”, and “cellular response to interleukin-1”. GO terms related to cell migration and proliferation, such as “chemotaxis” and “cell chemotaxis”, were also observed (Table S4).

On day 8, 1331 transcripts were differentially expressed, of which 508 were upregulated in the ASC-CM-treated samples, including ECM regulators, such as *MMP3* and *MMP1*. In contrast, 823 genes were downregulated, including genes involved in fibrosis, such as *CTGF* (Figure 6C). The GSEA revealed that DMEM-treated samples were enriched for “collagen fibril organization” (Figure 6D). The GO analysis of genes downregulated in ASC-CM-treated samples showed that in addition to inflammation-related GO terms, such as “immune response” and “inflammatory response”, there were also ECM-related GO terms, such as “skeletal system development”, “extracellular matrix organization”, and “epidermis development” (Table S5).

On day 14, 1106 transcripts were differentially expressed, of which 498 were upregulated in ASC-CM-treated samples and 608 were downregulated. The ASC-CM-downregulated transcripts included *ACTA2*, *TGF-b1*, *DKK2*, *COL1A1*, *COL11A1*, and *COL12A1* (Figure 6E). The GSEA indicated that gene sets such as “collagen fibril organization” and “external encapsulating structure organization” were enriched in DMEM-treated samples but not in ASC-CM-treated samples (Figure 6F). The GO analysis of the downregulated genes in ASC-CM-treated samples on day 14 showed GO terms related to the extracellular matrix, such as “cell adhesion”, “extracellular matrix organization”, “collagen fibril organization”, “skeletal system development”, and “collagen catabolic process”, instead of GO terms related to inflammatory response, as seen on days 4 and 8 (Table S6).

These data show that ASC-CM suppresses inflammation-related gene expression in the early phase of contraction; in the later phase, this suppression is gradually replaced by ECM-related gene expression.

### 3.7. Effect of ASC-CM on the Early Stage of KF Disc Formation

**Figure 7:**
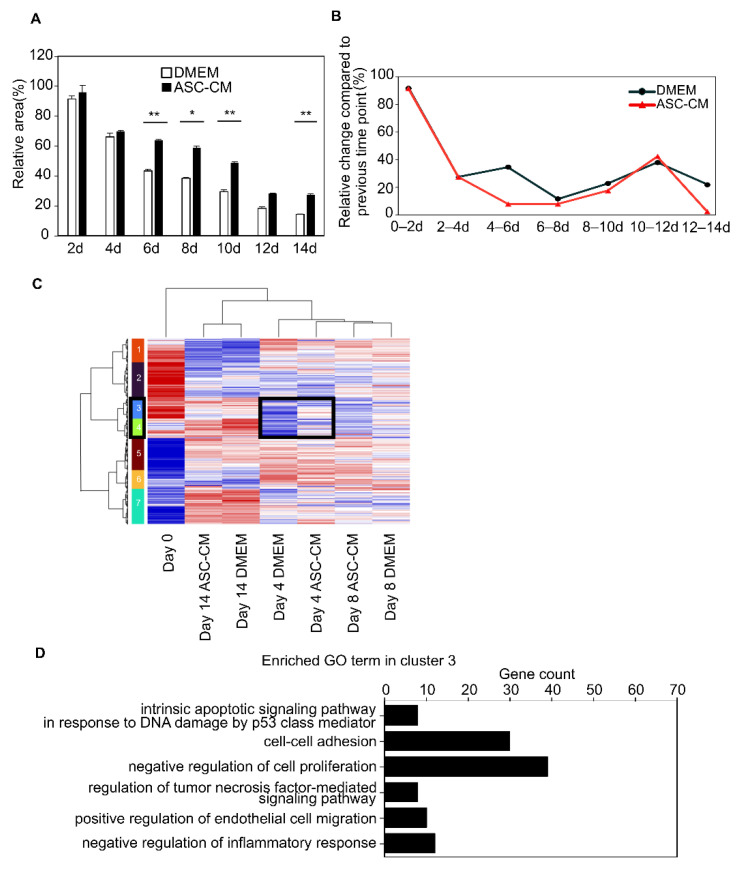
Effect of ASC-CM. (**A**) The relative contraction area was calculated as a percentage of the day 1 DMEM group. Results are presented as the mean ± standard deviation of six biological replicates. * *p* < 0.05, ** *p* < 0.01, unpaired Student’s *t*-test. (**B**) The rate of contraction compared to that of the previous time point. The most noticeable difference occurred between days 4 to 6. (**C**) Heatmap of hierarchical clustering. Clusters 3 and 4 (black square) had a large difference in gene expression on day 4. (**D**) GO terms of genes included in cluster 3. Abbreviations: ASC-CM, conditioned media derived from adipose stem cells; DMEM, Dulbecco’s modified Eagle’s medium; GO, Gene Ontology; RNA, ribonucleic acid; DNA, deoxyribonucleic acid.

Finally, we compared the disc area every 2 days to examine the details of the inhibitory mechanism of contraction induced by ASC-CM. The results showed no significant difference in the decrease rate on day 4 compared to day 1 between the samples. In contrast, there was a significant difference on day 6 and later between the DMEM and ASC-CM culture groups (Figure 7A). In addition, the rate of contraction over time was examined in the DMEM and ASC-CM culture groups. Notably, the DMEM group contracted from 66% to 43% of the initial area (contraction rate: 35%). In contrast, the ASC-CM group contracted only from 69% to 64% of the initial area (contraction rate: 8.0%). These results suggest that ASC-CM was most effective after day 4 (Figure 7B). In contrast, using hierarchical clustering of differentially expressed genes, the largest difference in gene expression was observed on day 14, followed by a large difference in gene expression on day 4 (Figure 7C, clusters 3 and 4). The GO analysis of cluster 3 and cluster 4 are shown in the (supplemental information Tables S7 and S8). Notably, cluster 3 contained 1062 genes, and the GO analysis with the top 10 lower *p*-values of these genes showed the terms “intrinsic apoptotic signaling pathway in response to DNA damage by p53 class mediator” and “negative regulation of cell proliferation”. In addition, there were “regulation of tumor necrosis factor-mediated signaling pathway” and “negative regulation of inflammatory response”, suggesting that the effect of ASC-CM on collagen disk contraction was due to attenuated cell proliferation and the suppression of the response to inflammatory stimulation. (Figure 7D, Table S7).

## 4. Discussion

MSCs are known to possess anti-inflammatory and immunomodulatory properties due to the paracrine action of their secretions, and they are thought to suppress scar formation, which results from excessive inflammatory reactions during wound healing [40,41] Research has been actively conducted on the application of ASCs in scar treatment [18]. However, basic research on treating pathological scars has been limited to studies of fibroblasts cultured in two-dimensional monolayers. Indeed, no appropriate animal models to mimic human scar tissue have yet been developed, and it has been challenging to demonstrate in vivo that ASCs improve scar tissue. In this study, we developed a model using a classical collagen contraction model with keloid-derived fibroblasts in the FPCL model.

The wound-healing cascade progresses in four phases: hemostasis, inflammation, proliferation, and remodeling. Fibroblasts are strongly involved in the latter three phases [42]. The FPCL model revealed gene expression related to ECM development and inflammatory response, starting with fibroblast proliferation. The progress of changes in gene expression was quite similar to that occurring during the wound healing process in living organisms; cell proliferation is followed by the activation of the inflammatory response, and finally, a gradual transition to the development and remodeling of the ECM. These data indicate that the FPCL model with keloid-derived fibroblasts can be used as an in vitro disease model for human scar tissue. However, this model is simple and comprises only collagen fibers and fibroblasts, rendering it inadequate for the reproducibility of in vivo biological skin tissue. Indeed, in addition to fibroblasts and collagen, wound healing involves a complex interplay between inflammatory cells such as macrophages and neutrophils; extracellular matrices other than collagen, such as glycosaminoglycans and hyaluronic acid; and cytokines supplied by epidermal cells, subcutaneous adipose tissue, and inflammatory cells [36]. Moreover, wound healing is also impacted by angiogenesis, as well as a myriad of other factors [7,15]. Hence, in the future, it will be necessary to devise a model that also accounts for these complex factors.

Previous studies have reported that gel contraction is enhanced in FPCLs supplemented with TGF-β [14,21], while the addition of PDGF results in gel contraction comparable to that observed in case of FPCLs with higher cell concentrations [43]. Similarly, we previously reported the fabrication of capillary-like structures after embedding vascular endothelial cells and MSCs in a perfusable system in a liver model [44].

ASCs and ASC culture supernatants have been used in regenerative medicine for various diseases [45,46,47]. As expected, ASC-CM exhibited a contractile inhibitory effect on scar contraction in this study. Wound contraction, which begins 2–3 days after wound infliction, is an advantageous mechanism for reducing the wound area and allowing the wound to heal quickly. However, this can lead to the exacerbation of keloid and hypertrophic scar formation by generating skin with insufficient extensibility or contracture, thus, limiting joint function. Hence, if ASC-CM can be applied onto, or injected into, the wound when wound contraction first begins, it may suppress excessive contraction during wound healing and reduce the risk of scar contracture and keloid formation. Future studies are expected to identify more effective time points in the progression of pathological scars and explore not only the treatment protocol of ASC-CM, but also the functional components contained in ASC-CM.

Moreover, as mentioned above, the FPCL model is insufficient as a scar model in terms of the immune response; therefore, a future innovative FPCL model is necessary. More specifically, it will be necessary to devise a model in which inflammatory cells, such as macrophages, are added to the models. In particular, given that we observed the reduced susceptibility of fibroblasts to inflammation after ASC-CM treatment, it will be necessary to develop a model containing macrophages and T cells to further elucidate the effects of inflammatory cytokines produced by these cells.

In addition, the present study only examined the natural contraction of collagen without considering the mechanical strain, which is closely related to the formation of pathological scars. In fact, mechanical strain on collagen gel increases the expression levels of stress-related genes, such as HSP27, and scar-related genes [48], while decreasing the expression levels of apoptosis-related genes [49]. Hence, future studies should examine the changes in gene expression and the effects of ASC-CM when mechanical strain is applied to the FPCL model. In addition to this in vitro system, it is also necessary to obtain pivotal data by analyzing organ cultures [50].

## 5. Conclusions

In this study, we reproduced scar contraction using a simple collagen contraction model and verified the efficacy of ASC-CM. Although the usefulness of this model and ASC-CM is limited to contraction, the mechanism of contraction and the action of ASC-CM are expected to be useful for the basic analysis of pathological scars and provide important evidence in plastic surgery.

## Data Availability

RNA-seq data are available in the DNA Data Bank of Japan (DDBJ) Sequence Read Archive under accession number DRA014100.

## Supplementary Materials

The following supporting information can be downloaded at: https://www.mdpi.com/article/10.3390/biomedicines10102388/s1; Table S1: Gene ontologies with the top 10 lowest *p*-values of DMEM treated samples on the day 4; Table S2: Gene ontologies with the top 10 lowest *p*-values of DMEM treated samples on the day 8; Table S3: Gene ontologies with the top 10 lowest *p*-values of DMEM treated samples on the day 14; Table S4: Gene ontologies with the top 10 lowest *p*-values depleted in ASC-CM compared to in DMEM at day 4; Table S5: Gene ontologies with the top 10 lowest *p*-values depleted in ASC-CM compared to in DMEM at day 8; Table S6: Gene ontologies with the top 10 lowest *p*-values depleted in ASC-CM compared to in DMEM at day 14; Table S7: Gene ontologies with the top 10 lowest *p*-values of cluster 3; Table S8: Gene ontologies with the top 10 lowest *p*-values of cluster 4.
