# Supplementary material for: Therapeutic Potential of Adipose Stem Cell-Derived Conditioned Medium on Scar Contraction Model
Source: Biomedicines. 2022 Sep 24;10(10):2388. doi: 10.3390/biomedicines10102388 (PMC9598573; doi:10.3390/biomedicines10102388)
Supplement: Supplementary file 1 [file biomedicines-10-02388-s001.zip › biomedicines-1896496-supplementary.pdf]

Table S1. Gene ontologies with the top 10 lowest *p*-values of DMEM treated samples on the day 4

**GOID**	**Description**	***p*-value**	**Genes symbol**
GO:0030593	neutrophil chemotaxis	8.89564E-06	*ITGA9, CCL7, TREM1, CCL20, PDE4B, CCL2, CXCL8, ITGA1*
GO:0006260	DNA replication	2.13387E-05	*MCM3, POLD3, TIMELESS, DBF4, RFC5, MCM4, RMI2, NASP, DNA2, RMI1, CCDC88A, BARD1, POLA1, BRIP1, MCM8, CLSPN, CDK2*
GO:0000731	DNA synthesis involved in DNA repair	7.78326E-05	*BARD1, POLD3, POLA1, RAD51B, BRIP1, RMI2, DNA2, RMI1*
GO:0006281	DNA repair	9.75857E-05	*SMC1A, MSH2, PARP2, RFC5, RECQL, SLF1, NABP1, PARPBP, UCHL5, RFWD3, POLA1, FANCL, RAD51B, POLQ, SMC3, FANCI, CLSPN, GEN1, DDX11, CDK2*
GO:0051301	cell division	0.000698835	*USP37, SMC1A, SPDL1, KIF14, CETN3, TIMELESS, CCSAP, SMC4, PMF1-BGLAP, NCAPG2, CENPF, NCAPD3, CCND1, ERCC6L, KIF20B, MASTL, NEDD1, CDC14A, CENPJ, SMC3, CDK2, CABLES2, MIS18BP1*
GO:0042060	wound healing	0.000714325	*ITGA9, IL24, WNT5A, PAK1, CCL20, SERPINB2, FGFR1OP2, TGFA, EREG*
GO:0000732	strand displacement	0.000979047	*BARD1, RAD51B, BRIP1, RMI2, DNA2, RMI1*
GO:0007099	centriole replication	0.001026411	*CEP152, CENPJ, CDK2, PLK4, SASS6*
GO:0007062	sister chromatid cohesion	0.001060729	*CENPI, CENPL, SMC1A, ERCC6L, SPDL1, CENPK, CENPQ, SMC3, PMF1-BGLAP, CENPF, DDX11*
GO:0000724	double-strand break repair via homologous recombination	0.001201353	*RAD54B, MMS22L, RAD51B, POLQ, SWSAP1, MCM8, GEN1, NABP1, RECQL*

Table S2. Gene ontologies with the top 10 lowest *p*-values of DMEM treated samples on the day 8

**GOID**	**Description**	***p*-value**	**Genes symbol**
GO:0071805	potassium ion transmembrane transport	0.000613	*KCNN4, SLC9A3, SLC9A5, KCNMB3, KCNAB1, KCNH5, KCNK3*
GO:0051056	regulation of small GTPase mediated signal transduction	0.002384	*NET1, FGD4, TIAM1, RAC3, ARHGAP22, ARAP2, NGEF, STARD8, ARHGAP39*
GO:0006955	immune response	0.006847	*TNFSF12-TNFSF13, TNFSF11, ENPP1, CIITA, HLA-B, IL15, TNFRSF6B, B2M, CHUK, JCHAIN*
GO:0050427	3'-phosphoadenosine 5'-phosphosulfate metabolic process	0.008382	*ENPP1, SULT1A3, SULT1B1*
GO:0051965	positive regulation of synapse assembly	0.009981	*EPHB1, NTRK2, LRRN3, LRRTM1, EFNA5*
GO:0030574	collagen catabolic process	0.017147	*COL11A2, ADAMTS3, COL6A6, MMP7, MMP8*
GO:0007130	synaptonemal complex assembly	0.01724	*SYCP2, SYCE1, REC8*
GO:0007631	feeding behavior	0.02869	*NEGR1, NTRK2, MRAP2*
GO:0030500	regulation of bone mineralization	0.033022	*PHOSPHO1, ENPP1, MGP*
GO:0006813	potassium ion transport	0.035327	*KCNN4, KCNMB3, KCNAB1, KCNK3*

Table S3. Gene ontologies with the top 10 lowest *p*-values of DMEM treated samples on the day 14

GOID	Description	*p*-value	Genes symbol
GO:0030198	extracellular matrix organization	3.37515E-06	*FOXF1, ITGA11, FBLN1, VIT, COL11A1, TNC, COL16A1, NID1, COL5A2, SMOC2, COL8A2, COL8A1, VCAM1, MFAP2, ITGB5, FBN1, PXDN, LAMC3*
GO:0007155	cell adhesion	3.86451E-06	*SEMA5A, ADAM17, ITGA11, COL12A1, CDH11, SPON2, PCDHB11, TNC, CD34, EPHA4, WISP2, NINJ2, THBS2, THBS4, VCAM1, MFGE8, LRFN3, PTPRS, CLCA2, COL16A1, COL8A1, ITGB5, MFAP4, ENTPD1, FBLN7, ROBO2, LAMC3*
GO:0007275	multicellular organism development	0.000187371	*ANGPTL2, ZNF516, TLL2, TMEFF1, DKK2, TNFRSF14, MITF, MC1R, TSNAXIP1, ZSWIM9, WNT11, SALL4, TSHZ2, ZFP41, PBXIP1, VANGL2, HIVEP1, FRAT2, PLXNA1, ROR2, PDGFD, TSHZ3, MXD1, CCDC120, SFRP2*
GO:0030335	positive regulation of cell migration	0.00098139	*SEMA5A, FOXF1, ADAM17, SEMA3E, CTSH, PDGFRB, ROR2, TCAF2, PDGFD, SEMA6C, GPER1, WNT11, EDN1, IRS2*
GO:0008284	positive regulation of cell proliferation	0.001327051	*IL7, PTH1R, ADAM17, HDAC4, CTSH, PDGFRB, FGFR2, TNC, FGFR1, AKR1C3, DPP4, IL34, TNS3, AKR1C2, PDGFD, GPER1, ALDH1A2, EDN1, TNFSF13B, IRS2, LRP5, SFRP2*
GO:0030326	embryonic limb morphogenesis	0.002477965	*SKI, SP9, MEGF8, SALL4, TBX5, FGFR1*
GO:0060349	bone morphogenesis	0.002798992	*SKI, PAPPA2, FGFR2, LRP5, SFRP2*
GO:0010875	positive regulation of cholesterol efflux	0.003363154	*PLTP, ABCA7, LRP1, ABCA1*
GO:0030574	collagen catabolic process	0.004203235	*COL8A2, MRC2, COL8A1, COL12A1, MMP15, COL11A1, COL5A2*
GO:0035987	endodermal cell differentiation	0.005777322	*COL8A1, ITGB5, COL12A1, MMP15, COL11A1*

Table S4. Gene ontologies with the top 10 lowest *p*-values depleted in ASC-CM compared to in DMEM at day 4

**GOID**	**Description**	***p*-value**	**Genes symbol**
GO:0006955	immune response	3.03967E-08	*IL32, CXCL6, OAS2, CCL2, LIF, THBS1, IL6, CXCL2, TNFSF12-TNFSF13, IL1RAP, CXCL1, TNFSF11, CXCL8*
GO:0070098	chemokine-mediated signaling pathway	6.34777E-07	*CXCL6, CCL2, ACKR3, CXCL2, CXCL1, CXCL8*
GO:0071356	cellular response to tumor necrosis factor	3.12186E-06	*CCL2, ANKRD1, THBS1, VCAM1, IL6, ASS1, CXCL8, EDN1*
GO:0045429	positive regulation of nitric oxide biosynthetic process	7.17706E-06	*PTGS2, AGT, PTX3, IL6, ASS1, EDN1*
GO:0006935	chemotaxis	1.61191E-05	*CXCL6, CCL2, ACKR3, CYR61, CXCL2, CXCL1, CXCL8*
GO:0006954	inflammatory response	3.04927E-05	*CXCL6, CCL2, TSPAN2, THBS1, PTGS2, PTX3, IL6, CXCL2, IL1RAP, CXCL1, CXCL8*
GO:0060326	cell chemotaxis	0.000356406	*CXCL6, CCL2, VCAM1, CXCL2, CXCL1*
GO:0071347	cellular response to interleukin-1	0.000356406	*CCL2, ANKRD1, IL6, CXCL8, EDN1*
GO:0007155	cell adhesion	0.000415505	*IL32, HAPLN1, CCL2, ACKR3, THBS1, OMD, CTGF, PCDHA3, VCAM1, CYR61, CD24*
GO:0048661	positive regulation of smooth muscle cell proliferation	0.000896931	*NR4A3, THBS1, PTGS2, IL6, EDN1*

Table S5. Gene ontologies with the top 10 lowest *p*-values depleted in ASC-CM compared to in DMEM at day 8

**GOID**	**Description**	***p*-value**	**Genes symbol**
GO:0001501	skeletal system development	1.44637E-08	*COL10A1, HAPLN1, IGF2, CMKLR1, COMP, TNFRSF11B, COL1A1, CHRD, PHEX, RASSF2, IGF1, PKDCC, MATN3*
GO:0030198	extracellular matrix organization	5.88166E-06	*COL10A1, HAPLN1, ELN, TGFBI, COL9A3, THBS1, COL5A1, COMP, ECM2, COL1A1, MATN3, TNFRSF11B*
GO:0030282	bone mineralization	4.12692E-05	*PTN, PHEX, ASPN, TUFT1, PKDCC, CLEC3B*
GO:0007155	cell adhesion	8.86362E-05	*HAPLN1, IL32, APLP1, NEDD9, OMD, EFNB2, COL5A1, EPHA3, COL15A1, COMP, COL1A1, TGFBI, THBS1, CTGF, MFAP4*
GO:0006955	immune response	0.000526688	*IL32, CMKLR1, CRIP1, THBS1, IL16, CXCL2, IL1RAP, TNFRSF11B, MAP3K14, TNFRSF6B*
GO:0008544	epidermis development	0.001039465	*CRABP2, CTGF, KRT16, KRT34, KRT14*
GO:0006954	inflammatory response	0.001066716	*TSPAN2, THBS1, PLA2G4B, KRT16, C4A, PTX3, CXCL2, IL1RAP, TRIL, TNFRSF11B, TNFRSF6B*
GO:0044267	cellular protein metabolic process	0.0012618	*IGF2, TGFBI, PGA3, ADORA2B, PAPPA2, IGF1*
GO:0006508	proteolysis	0.002863469	*PHEX, PGA3, ADAMTS15, XPNPEP2, HTRA3, PAPPA2, UFSP1, C4A, ADAMTS10, MASP1, ADAMTS16*
GO:0061436	establishment of skin barrier	0.002904707	*FLG, KRT16, TMEM79*

Table S6. Gene ontologies with the top 10 lowest *p*-values depleted in ASC-CM compared to in DMEM at day 14

GOID	Description	*p*-value	Genes symbol
GO:0007155	cell adhesion	8.30973E-19	*HAPLN1, NEDD9, LOXL2, COL16A1, EFNB2, COL5A1, TINAGL1, ITGBL1, BCAM, ITGB2, THBS1, CTGF, COL8A1, TNC, SCARF1, LAMC3, PKP1, COL12A1, ROBO2, AOC3, HES1, COL15A1, RHOB, COMP, CD34, COL1A1, THBS4, GP1BB, DPT, TGFBI, ATP1B1, VCAM1, SPON1, MFAP4*
GO:0030198	extracellular matrix organization	1.2084E-13	*COL10A1, HAPLN1, LAMC3, COL16A1, COL5A1, HSPG2, SPARC, COMP, COL1A1, BGN, ELN, LOX, TGFBI, ITGB2, THBS1, COL8A1, COL8A2, VCAM1, COL11A1, MATN3, TNC*
GO:0030199	collagen fibril organization	1.43908E-06	*LOXL2, LOX, DPT, COL5A1, SERPINF2, COL12A1, COL11A1, COL1A1*
GO:0001501	skeletal system development	4.25762E-06	*COL10A1, HAPLN1, GDF10, CMKLR1, EXTL1, COL12A1, IGF1, COMP, COL1A1, MATN3, NPR3*
GO:0030574	collagen catabolic process	1.07783E-05	*COL10A1, COL5A1, COL8A2, COL8A1, COL12A1, COL15A1, COL11A1, COL1A1*
GO:0032870	cellular response to hormone stimulus	9.03104E-05	*SIK1, FOSB, ROBO2, SIK1B, SLIT3, OXTR*
GO:0071300	cellular response to retinoic acid	0.000179929	*WNT7B, CYP26B1, BRINP2, AQP1, WNT2, COL1A1, TNC*
GO:0030324	lung development	0.000300014	*WNT7B, LOX, CTGF, WNT2, HES1, SPARC, FGF18*
GO:0045721	negative regulation of gluconeogenesis	0.000457811	*SIK1, C1QTNF3, IL6, SIK1B*
GO:0007568	aging	0.000757986	*LOXL2, UCP2, ITGB2, DMD, NTRK1, CTGF, VCAM1, IL6, KRT14*

Table S7. Gene ontologies with the top 10 lowest *p*-values of cluster3

GOID	Description	*p*-value	Genes symbol
GO:0006366	transcription from RNA polymerase II promoter	8.60E-05	*AKNA, CAMTA2, CEBPB, CEBPD, DBP, EGR1, EGR2, ELF4, ELK1, EPAS1, ETS1, FOS, GMEB2, GTF2F1, IRF1, IRF3, IRF7, JUND, KLF4, MAFK, MED16, MEIS3, MLLT1, MSC, NELFB, NFATC1, NFIC, NRL, OSR2, PAX3, PBX4, PIR, PITX1, POLR2A, POLR2E, POLR2J3-UPK3BL2, SALL4, SCAF1, SOX12, SSBP4, SUPT5H, TAF6, TFAP2A, TP53, USF2*
GO:0000122	negative regulation of transcription from RNA polymerase II promoter	8.66E-04	*AJUBA, ANKRD2, ARID5B, BCL11A, BHLHE41, BTG2, CBX4, CBX8, CC2D1A, CD36, CRY2, DACT1, EDN1, EGR1, ERF, ESRRA, FST, HDAC5, HEXIM2, HEY2, HSF1, IFI27, IRF7, JDP2, JUND, KLF4, LMCD1, MBD3, MEN1, MEPCE, MSC, NCOR2, NFIC, NR1H2, NR2F6, OSR2, PCGF2, PIAS4, PKIG, PPARD, PPP1R13L, RARA, RARG, RBM10, RELB, RITA1, SALL4, SMAD3, STAT3, SUPT5H, TFAP2A, TGIF1, TLE4, TLE5, TP53, TRIM28, VAX2, WFS1, YBX3, ZFP36, ZFPM1, ZNF296*
GO:0042771	intrinsic apoptotic signaling pathway in response to DNA damage by p53 class mediator	0.001032	*BAG6, CDKN1A, NUPR1, PHLDA3, PML, PYCARD, SHISA5, TP53*
GO:0098609	cell-cell adhesion	0.001637	*ARFIP2, ARHGAP1, ATXN2L, BAIAP2, CC2D1A, CCS, CORO1B, DBN1, EHD1, EPHA2, EPS8L2, FLRT3, FSCN1, GIPC1, H1-10, HSPA1A, MARK2, MICALL1, MPP7, PLIN3, PPP1R13L, RAB11B, SCYL1, SEPTIN9, SH3GL1, STK38, TBC1D10A, TWF2, UNC45A, VASN*
GO:0033574	response to testosterone	0.002503	*BAD, DUSP1, EDN1, HSF1, PLN, TBXA2R, THBS1, TSPO*
GO:0008285	negative regulation of cell proliferation	0.00414	*ADARB1, ADM, B4GALT1, BAK1, BTG2, CDKN1A, CSK, CXCL1, ETS1, FGFRL1, FUZ, GSDME, HSF1, IFITM1, IRF1, KLF4, MEN1, NACC2, NTRK1, P3H1, P3H3, PLAAT4, PML, PTK2B, QSOX1, RARA, RARG, RBM10, RBM38, RGCC, RPS6KA2, SIRT6, SLIT3, STAT3, TAF6, TFAP2A, TNS2, TP53, TP53I11*
GO:0010803	regulation of tumor necrosis factor-mediated signaling pathway	0.00521	*CLIP3, IKBKG, PYCARD, RBCK1, RNF31, SHARPIN, TNFRSF1A, TRADD*
GO:0010595	positive regulation of endothelial cell migration	0.005892	*AGT, ANXA3, BCAR1, BCAS3, EDN1, ETS1, PRKD2, PTK2B, THBS1, ZNF580*
GO:0050728	negative regulation of inflammatory response	0.006739	*ETS1, FEM1A, KLF4, NLRX1, PPARD, PTGIS, SHARPIN, SMAD3, SOCS3, TNFAIP6, TNFRSF1A, ZFP36*
GO:0006977	DNA damage response, signal transduction by p53 class mediator resulting in cell cycle arrest	0.007206	*BTG2, CARM1, CDKN1A, GADD45A, MUC1, PLK2, PML, RBM38, RGCC, TP53, ZNF385A*

Table S8. Gene ontologies with the top 10 lowest *p*-values of cluster4

**GOID**	**Description**	***p*-value**	**Genes symbol**
GO:0007409	axonogenesis	1.45E-04	*CNP, ECM2, FGFR2, LLGL1, LRFN3, LRFN4, LUM, NOTCH1, NTNG2, OGN, OMD, PAK3, PRELP, STK11*
GO:0061337	cardiac conduction	4.93E-04	*CACNA1C, CACNB1, CACNB3, CACNB4, KCND2, KCNJ12, KCNK15, SCN1B*
GO:0007155	cell adhesion	7.31E-04	*ADAM12, CCN4, CCN5, CD34, CDH11, CLSTN1, CNTN1, COL12A1, COL8A1, CYP1B1, EFNB1, EMILIN1, EMILIN2, ENTPD1, EPHB4, GAS6, GP1BB, ITGA11, JUP, LAMB2, LGALS3BP, LRFN3, MFAP4, MFGE8, NECTIN4, NINJ2, NUAK1, OMD, PGAP6, PTK7, ROBO2, SCN1B, SEMA5A, SPON2, SVEP1, THBS2, THBS3, THBS4, TNXB, VCAM1*
GO:0008203	cholesterol metabolic process	0.001019	*APOE, CEBPA, CUBN, CYP7B1, LIPC, LRP5, PCSK9, RXRA, SCAP, SREBF1, SREBF2*
GO:0000122	negative regulation of transcription from RNA polymerase II promoter	0.002705	*ALX1, CBX6, CDKN1C, CEBPA, CIC, CREBBP, CXXC5, E4F1, FGFR1, FGFR2, FOXF1, FRK, GLIS1, GLIS2, HCFC1, HDAC4, HDAC7, HES6, HIC1, IRF2BPL, MDFI, MSX1, MYPOP, MZF1, NFIB, NFIX, NOTCH1, NR1D1, NR1H3, PLK3, RERE, RXRA, SKI, SKOR1, SMO, SREBF1, SREBF2, TCF3, THRB, TSHZ3, TXNIP, VDR, VHL, WDTC1, ZBTB20, ZBTB4, ZBTB7A, ZHX2, ZNF205, ZNF219, ZNF496*
GO:0042157	lipoprotein metabolic process	0.003246	*APOE, APOL3, APOL4, BMP1, CUBN, PCSK9, SDC1*
GO:0060484	lung-associated mesenchyme development	0.003682	*FGFR1, FGFR2, PTK7, WNT11*
GO:0010508	positive regulation of autophagy	0.005669	*PIM2, PLEKHF1, STK11, TFEB, TICAM1, TPCN1, TRIM8, ULK1*
GO:1900182	positive regulation of protein localization to nucleus	0.00639	*CDKN2A, GLIS2, NGFR, SRC, STK11, TRIM8*
GO:0030514	negative regulation of BMP signaling pathway	0.007327	*ABL1, BMPER, CHRDL1, HTRA3, LEMD2, NOTCH1, SKI, SKOR1*
